# Comments on oral lesions and disorders and their prevalence arising from the use of illicit drugs in a prison population

**DOI:** 10.2340/aos.v84.43601

**Published:** 2025-05-13

**Authors:** Manas Bajpai

**Affiliations:** Department of Oral Pathology and Microbiology, Rural Dental College, Loni (Maharashtra) India

To the editor,

With great interest, I read the article titled ‘Oral lesions and disorders and their prevalence arising from the use of illicit drugs in a prison population’ by Relvasa et al., published in Acta Odontologica Scandinavica. I found this article enlightening and up to the minute [1]. It provides a valuable contribution to understanding the impact of illicit drug use on oral health within a particularly vulnerable and often overlooked population: prisoners. The primary strength of this study lies in its focus on a neglected population. Prison populations often face significant barriers to healthcare, including dental care. Understanding the specific oral health challenges associated with illicit drug use within this context is crucial for developing targeted interventions and improving overall health outcomes.

The study likely employs a cross-sectional design, which limits the ability to establish causal relationships between illicit drug use and oral health outcomes [2]. It is possible that other factors, such as poor diet, a lack of access to oral hygiene products, or co-existing medical conditions, contribute to the observed oral lesions and disorders. If the study relies on self-reported drug use, there is a potential for underreporting due to social desirability bias or fear of repercussions. This could underestimate the true prevalence of drug-related oral health problems. Ideally, biochemical confirmation of drug use would be included. The article may not have specified which drugs are more related to the oral problems identified. Drug-related oral problems may vary depending on the type of drug, the route of administration, and duration of drug use.

Longitudinal studies are needed to establish temporal relationships between drug use and the development of oral lesions and disorders. Following a cohort of prisoners over time and tracking their drug use and oral health status would provide more robust evidence for causality. Future studies should incorporate objective measures of drug use, such as urine or hair follicle drug testing, to validate self-reported data and reduce the potential for bias [3]. Research should explore the specific biological mechanisms by which different drugs affect oral tissues. This could involve in vitro studies examining the effects of drugs on oral cells or in vivo studies examining the effects on experimental animal. While the study has limitations, it highlights a critical need for further research and intervention efforts. By addressing the limitations identified in this commentary and pursuing the suggested avenues for future research, we can improve the oral health and overall well-being of this vulnerable population. The study serves as a call to action for oral health professionals, public health advocates, and policymakers to prioritize the oral health needs of prisoners and integrate oral healthcare into comprehensive addiction treatment strategies.
